# Mycorrhizae set the stage for plants to produce a higher production of biomolecules and stress-related metabolites: a sustainable alternative of agrochemicals to enhance the quality and yield of beetroot (*Beta vulgaris L*.)

**DOI:** 10.3389/fmicb.2023.1196101

**Published:** 2023-07-03

**Authors:** Vinod Kumar Yadav, Deepesh Kumar, Radha Krishna Jha, Rakesh Kumar Bairwa, Rajan Singh, Gaurav Mishra, Jyoti Prakash Singh, Adarsh Kumar, Banoth Vinesh, Kuldip Jayaswall, Abhishek Kumar Rai, Arvind Nath Singh, Sanjay Kumar, Mahendra Vikram Singh Rajavat, Deepanshu Jayaswal

**Affiliations:** ^1^University Department of Botany, Ranchi University, Ranchi, Jharkhand, India; ^2^ICAR-National Institute for Plant Biotechnology, New Delhi, India; ^3^ICAR-Indian Institute of Wheat and Barley Research, Karnal, India; ^4^ICAR-Indian Institute of Vegetable Research, Varanasi, India; ^5^Chandra Shekhar Azad University of Agriculture and Technology, Kanpur, India; ^6^ICAR-National Bureau of Agriculturally Important Microorganism, Mau, Uttar Pradesh, India; ^7^ICAR-Indian Institute of Seed Science, Mau, Uttar Pradesh, India

**Keywords:** ALDH7B4, ALDH3I1, beetroot, *Glomus mosseae*, *Gigaspora gigantea*, mycorrhizal inoculation, *Acaulospora laevis*, soil

## Abstract

Population explosions, environmental deprivation, and industrial expansion led to an imbalanced agricultural system. Non-judicial uses of agrochemicals have decreased agrodiversity, degraded agroecosystems, and increased the cost of farming. In this scenario, a sustainable agriculture system could play a crucial role; however, it needs rigorous study to understand the biological interfaces within agroecosystems. Among the various biological components with respect to agriculture, mycorrhizae could be a potential candidate. Most agricultural crops are symbiotic with arbuscular mycorrhizal fungi (AMF). In this study, beetroot has been chose to study the effect of different AMFs on various parameters such as morphological traits, biochemical attributes, and gene expression analysis (ALDH7B4 and ALDH3I1). The AMF Gm-*Funneliformis mosseae* (*Glomus mosseae*), *Acaulospora laevis*, and GG-*Gigaspora gigantean* were taken as treatments to study the effect on the above-mentioned parameters in beetroot. We observed that among all the possible combinations of mycorrhizae, Gm+Al+GG performed best, and the Al-alone treatment was found to be a poor performer with respect to all the studied parameters. This study concluded that the more the combinations of mycorrhizae, the better the results will be. However, the phenomenon depends on the receptivity, infectivity, and past nutrient profile of the soil.

## Introduction

The increasing population worldwide has created huge pressure on the agriculture system due to the high demand for grain for pulses, vegetables, and fruits. During the green revolution in the 1960s, several chemical fertilizers, pesticides, and herbicides were used to meet the high demand for food (Singh, 2000). The ultimate result was increased agricultural production with the uncontrolled use of agrochemicals led to the imbalanced bio-physiochemical properties of the soil. In the current scenario, when climate change is the major cause of several biotic and abiotic stresses, a second green revolution in a sustainable way is the need of the hour (Bhatt et al., 2016). The application of biofertilizer instead of chemical fertilizer could be the major contributor to attaining the second green revolution.

Vegetables, particularly beetroot (*Beta vulgaris* L.), are being used in the food, pharmaceutical, and sugar industries (El-Beltagi et al., 2022). Considering the yield and quality parameters, it is clearly evident that beetroot produced without agrochemicals is highly desirable for human consumption, which could be achieved using biological organisms, such as mycorrhizae and other soil bacteria, without compromising the yield and quality of the beetroot. Beetroot is a biennial flowering plant that belongs to the Amaranthaceae family and originated in Asia and Europe (Avetisyan et al., 2022; Thakur and Singh, 2022). Approximately 1,400 species in 105 genera of beetroot have been reported worldwide to date (Lazǎr et al., 2021). Looking at the nutrient profile of the beetroot, it contains a variety of biomolecules and active ingredients such as highly active pigments, dietary fibers, vitamins, and secondary metabolites including carotenoids, polyphenols, flavonoids, and saponins (Chhikara et al., 2019). The multiple biomolecules and secondary metabolites of the beetroot play a significant role as antioxidants, anti-inflammatory agents, anti-cancer agents, and anti-diabetic agents. It is reported that these active ingredients can help reduce cardiovascular disease, promote wound healing, and provide several other health benefits (Sun and Lu, 2019).

While going through the health benefits of beetroot, several studies were carried out focusing on its growth and development, crop improvement through conventional and molecular breeding, nutrient profiling, cell suspension culture, and metabolic profiling, along with its regulation (Abu-Ellail et al., 2021; Carrera et al., 2021; Wikandari et al., 2021; Wang et al., 2022). The underutilized part of getting the maximum yield in a sustainable way is the application of bioprospecting. Therefore, the study of novel mycorrhizae to observe their effect on the physiological and biochemical aspects of beetroot is crucial. In some studies, it is reported that mycorrhizae, particularly arbuscular mycorrhizal fungi (AMF), could be used to enhance plant growth, biotic and abiotic stress tolerance, and ultimately productivity (Khaliq et al., 2022). The AMF is among the best examples of obligate symbionts, where the fungal partner provides water to the plant and absorbs nutrients from the soil for the host (Manga et al., 2022). In an agricultural context, AMF alters the soil structure by crosslinking highly dense mycelium with soil particles, which reduces soil erosion, and enhances soil water retention capacity (Leifheit et al., 2014). According to Van Der Heijden et al. (2006), AMF do not directly increase plant productivity; rather, they significantly help in phosphorus and nitrogen acquisition and the survival of different plant species. The environmental conditions and availability of phosphorus and nitrogen in soil affect the successful colonization of AMF and symbiosis (Baar, 2008).

The AMF are of key importance for the plant's physiology, *viz*., the biomass of the root, osmotic balance, mineral contents, chlorophyll content, stomatal conductance, photosynthetic, and respiration rates, as well as antioxidant activities (Liu et al., 2014; Pedranzani et al., 2016). The resulting effects of these AMF on plants are increased nutritional content and rapid plant growth (Jabborova et al., 2021a,b). In this line, several researchers have concluded that mycorrhizae alone and in combination with other microorganisms could be used in the agriculture sector to enhance crop yield by modifying plant physiology, including height, leaf, and root parameters (Mathur and Sharma, 2018). Recent studies on the effect of AMF on the physiology of several crops have proven that AMF plays a significant role in increasing the total chlorophyll, carotenoid, nutrient, phytohormones, antioxidants, SODs, and peroxidases, ultimately affecting the overall metabolism of the plants, and resulting in high yields from the concerned crops (Basu et al., 2021; Jabborova et al., 2021c, 2022; Jabborova, 2022).

Therefore, in this study, we proposed the hypothesis that bioinoculants (Gm-*Funneliformis mosseae*, Al-*Acaulospora laevis*, and G_G_-*Gigaspora gigantean)* act on (i) morphological and food storage parameters of *Beta vulgaris;* (ii) biochemical and physiological attributes of *Beta vulgaris;* (iii) stress physiological attributes of *Beta vulgaris;* (iv) mineral content of *Beta vulgaris;* and (v) aldehyde dehydrogenase gene expression.

## Materials and methods

### Field preparation and selection of beetroot cultivar

Before initiating the seed sowing and microbial treatment, the net house conditions were optimized. The desired conditions of 24°C and 49–66% relative humidity were established in the greenhouse. The soil was prepared by making a mixture of alluvial soil and sand in a ratio of 3:1 with 70.8% sand, 24.5% silt, and 4.0% clay. The chemical composition of the soil was analyzed (0.042% N, 0.017% P, 0.06% organic carbon, and a pH of 7.4) and found to be suitable for the cultivation of beetroot. Furthermore, the soil was autoclaved at 121°C to eliminate the seeds of weeds and microbial inoculums. The beetroot cultivar Crimson Globe was taken for sowing in pots during the month of October 2020. Furthermore, a randomized complete block design was chosen to give the desired treatments in triplicate, with 15 plants in each replication. The agronomical practices were followed by Yadav et al. (2021).

### Microbial inoculums preparation and treatments on beetroot

In this study, three microbial inoculums were taken for the treatment of beetroot in different combinations. The inoculum of *Funneliformis mosseae* (Gm) with 80–86% colonization (root pieces) and 780–800 AM spores (w/w) was procured from the Department of Botany, Kurukshetra University, Kurukshetra. The inoculum of *Gigaspora gigantea* (Gg) with 75–79% colonization (root pieces) and 870–890 AM spores (w/w) and *Acaulospora laevis* was procured from the Forest Pathology Discipline, Forest Protection Division, FRI, Dehradun, India. To get the starter inoculum for the experiment, both inoculums (*Funneliformis mosseae* and *Gigaspora gigantea*) were then mass multiplied using maize as a host for 3 months. After mass production, the inoculum containing 77–82% colonization/infection (maize root pieces) and 820–860 *Funneliformis mosseae* spores (w/w), and 74–78% colonization/infection (maize root pieces) and 840–880 *Gigaspora gigantea* spores (w/w) were taken. *Acaulospora laevis* was multiplied in a nutrient broth medium containing beef extract, peptone, and NaCl at 3 g/L, 5 g/L, and 5 g/L, respectively, and incubated at 32°C for 48 h for proper growth of AMF.

In total, 10 g of each inoculum was added per pot for a single treatment, and 5 + 5 g (*Funneliformis mosseae* + *Gigaspora gigantea*) was added for dual and consortium treatments. For the *Acaulospora laevis* treatment, all the seeds of beetroot except the control were dipped in the nutrient broth medium for 10 min (Saini et al., 2019). Microbial inoculums used in this study are shown in Table 1. The following seven treatments (Tt) were investigated to inoculate beetroot alongside the control.

**Table 1:** Treatments with single and different microbial combinations given to beetroot along with control.

**Treatment**	**Microbial inoculation**
C	Control
T1	Funneliformis mosseae (Gm)
T2	Acaulospora laevis (Al)
T3	Gigaspora gigantea (GG)
T4	Gm + Al
T5	Gm + GG
T6	Al + GG
T7	Gm + Al + GG (consortium)

### Morphological and food storage parameters of beetroot

**Figure 1:**
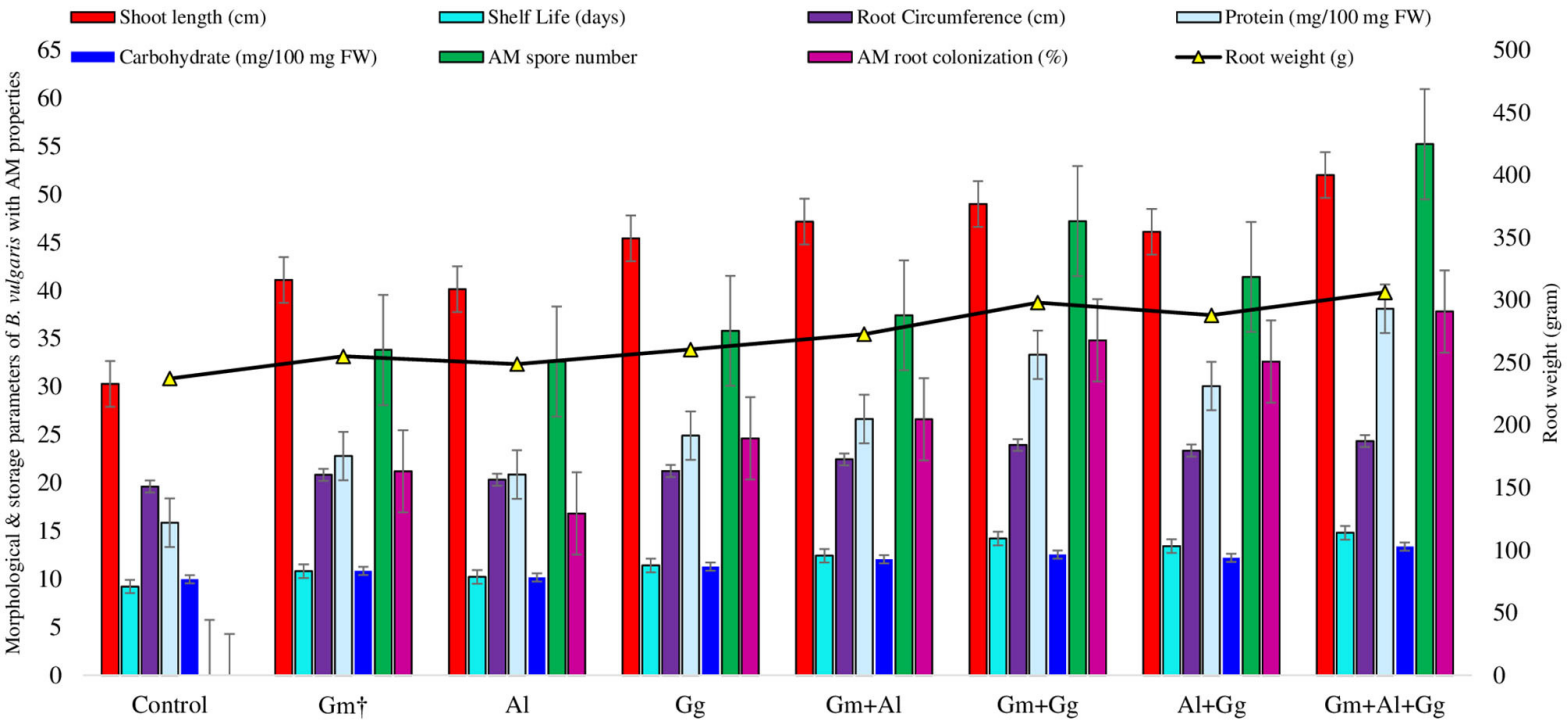
Morphological and food storage parameters of beetroot with AMF properties.

With all the treatments alone and in combinations, morphological and food storage parameter data, such as shoot length (cm), root weight (g), shelf life (days), root circumference (cm), protein (mg/100 mg FW), and carbohydrate (mg/100 mg FW), were recorded and statistically analyzed (Figure 1, Supplementary Table 1). To analyze the AMF colonization, beetroot roots were washed with water and chopped into small pieces. The digestion and clearance were carried out in KOH and stained with trypan blue (0.05%). Thereafter, AMF colonization was measured with a microscope. Based on the fungal hyphal infection, the frequency distribution method was followed to study the colonization (number of arbuscules) in the beetroot segments (Phillips, 1970; Giovannetti, 1980; Biermann, 1981; Koske, 1989).

### Biochemical and physiological attributes of beetroot

Several biochemical tests were performed (50 days after sowing) to measure total chlorophyll (mgFW-g), total anthocyanin (mgFW-g), phosphorus content (%) in root and shoot, and phosphatase (IU/g FW) from beetroot samples in triplicates (Supplementary Table 2). The absorbance of the supernatant was recorded at 645 nm and 663 nm for chlorophyll a and b, respectively, using a UV–vis spectrophotometer (Specord 205 Analytik Jena AG, Jena, Germany). Anthocyanin was measured at 520 nm using acetone (80%) as a blank (McGonigle et al., 1990). The recorded data were analyzed statistically using SPSS (version 11.5), and inferences were made.

### Measurement of stress-related attributes and mineral contents of beetroot

The peroxide content, electrolyte leakage, proline, catalase, ascorbate peroxidase, and superoxide dismutase (SOD) were measured following the protocol (Plenchette et al., 2005). The recorded data were statistically analyzed (Supplementary Table 3). In the control plants, individual inoculum-treated plants, consortium-treated plants, mineral levels, such as potassium (K), calcium (Ca), magnesium (Mg), iron (Fe), and manganese (Mn), were measured (Mitchell and Bingham, 1974; Kurt and Kizildag, 2018; Saini et al., 2020).

### RNA isolation and cDNA synthesis

The fresh leaf samples from all the treatments and control plants were procured in liquid nitrogen. The leaf samples were collected after 3 days of treatment. Total RNA was isolated from all the collected leaf samples using the Trizol method. The DNase (RNase-free) treatment was given to the isolated total RNA. The quantification of isolated RNA was performed by Nanodrop (Thermo Scientific Nanodrop 2000) along with a quality check by 1.2% denaturing agarose gel electrophoresis. For cDNA synthesis, the Thermo Scientific cDNA synthesis kit was used as per the protocol given by the manufacturers. All the samples were collected in triplicate.

### Gene expression analysis using *Arabidopsis thaliana* aldehyde dehydrogenase 7B4 and *Arabidopsis thaliana* aldehyde dehydrogenase 3I1

The primers for both the genes ALDH7B4 and ALDH3I1 (Table 2) were designed from *Arabidopsis thaliana* transcripts NM¬_179476.3:121-1647 and NM¬_001342263.1:749-1975, respectively, using IDT software.

**Table 2:** Primer sequences of ALDH7B4, ALDH3I1 used for gene expression analysis.

**S.N**.	**Genes**	**Primer sequence**
1	*ALDH7B4*	Forward: AAGCAGTCGAAGGTGAAGGA
		Reverse: GTTTTCAGGGTTCCGAGTGA
2	*ALDH3I1*	Forward: GAGGGAGGAGTCCCTGAAAC
		Reverse: TCCTCCTAGCAGCCACTTGT
3	*Actin*	Forward: TGTGCCAATCTACGAGGGTT
		Reverse: ACAACGGCACTACTGGATCA

The PCR reaction was set up in a 96-well PCR plate containing 2 μl of template, 0.5 μl primers, 10 μl SYBR green, and DEPC water. The qRTPCR reaction condition was denaturation at 94 °C for 3 min, 35 cycles of 94 °C for 30 s, 60 °C for 20 s, and 72 °C for 30 s. Actin was used as an internal control. Three technical replicates were taken for each sample, and “the comparative Ct method” was used to calculate the relative expression of each sample.

## Result and discussion

Environmental degradation, population explosion, and industrial globalization have created a situation in which we have to reconsider the agricultural system *via* restoration and reclamation of soil microbiota, bio-prospecting, and cutting-edge technology (Ortas, 2012). The unjudicial use of chemical fertilizers and pesticides led to an environmental loss, an unbalanced agroecosystem, and non-profitable crop cultivation. Before replacing the current system of agriculture with a sustainable agriculture system, we have to understand the deep biological interactions among plant and soil microbiota (Chun et al., 2018). With the conclusion of previous studies, it is now well known that mycorrhizae, which are symbiotic associations between fungi and plant roots, are very helpful in biotic and abiotic stress tolerance and mineral absorption, ultimately assisting in the enhanced productivity of various crops (Amirnia et al., 2019; Adeyemi et al., 2020; Wang et al., 2020). The AMF positively affects plant growth by balancing ROS levels in the cells, improving nutrient uptake by altering the root architecture, and increasing antioxidant levels (Augé, 2001; He et al., 2020). The AMF symbiosis also regulates the homeostasis of hormones in the plants that are responsible for changes in their physiology (Pozo et al., 2015). Due to AMF, the increasing amount of minerals, such as P, regulates gas exchange, transpiration, and drought stress (Pozo et al., 2015). Even though the importance of mycorrhizae in agricultural systems is well established, their implementation is very rare. Therefore, it is the need of the hour to do rigorous research on interactions between mycorrhizae and different crop roots, infectivity, and receptivity of mycorrhizae to the soil. With this background, we have tried to analyze our hypothesis on beetroot.

### Effect of bioinoculants on morphological and food storage parameters of beetroot

The morphological, biochemical, shelf life (days), and AMF root colonization (%) data in control and treated beetroot were recorded and analyzed (Figure 1, Supplementary Table 1). The shoot length of the beetroot was the highest, i.e., 51.974 cm, in the combination of treatments having G_m_- *Funneliformis mosseae*, Al-*Acaulo sporalaevis*, and G_G_-*Gigaspora gigantean*, followed by 48.974 cm in the treatment having G_m_+G_G_, 47.148 cm in G_m_+A_l_, 45.426 cm in G_G_, 40.118 cm in A_l_, 41.088 cm in Gm, and 30.280 cm in the control (Supplementary Table 1). The highest root weight was recorded in the treatment having Gm+Al+GG, i.e., 306.102 grams, and the least in Al, i.e., 248.716 grams (Figure 1). The highest root circumference was observed in the G_m_+A_l_+G_G_ treatment, i.e., 24.320 cm, and the least was in the A_l_ treatment at 20.310 cm (Supplementary Table 1). The shelf life of the beetroot was found to be the highest in the G_m_+A_l_+G_G_ treatment and the lowest in the A_l_ treatment. Looking into the nutrient profiling, i.e., protein and carbohydrate (mg/100 mg fresh weight), the highest protein and carbohydrate were observed in the G_m_+A_l_+G_G_ treatment, i.e., 38.092 and 13.352, respectively, and the least was in Al, i.e., 20.841 and 10.142, respectively (Supplementary Table 1). In this sequence, following the same pattern, AM spore number and AM root colonization (%) were the highest in the G_m_+A_l_+G_G_ treatment, i.e., 55.200 and 37.800, respectively, and the lowest in the Al treatment, i.e., 32.600 and 16.800, respectively. The values recorded for all the parameters were found to be the lowest in the control plants (Figure 1). The mentioned values related to all the parameters are given in Supplementary Table 1.

Our findings suggested that among the single and combinations of mycorrhizal treatments, the G_m_+A_l_+G_G_ treatment showed significant results for all the parameters. The most probable reason would be the synergistic effect of the mycorrhizae on beetroot. In most of the parameters, the performance of the A_l_ treatment was the lowest among all the treatments. This would be because the soil has less mycorrhizal infectivity and/or receptivity. As the control plants showed the least value for all the parameters, it could be because of the low or unbalanced nutrient profile of the soil. With this observation, it may be concluded that a more compatible combination of mycorrhizae could lead to an increased value of the studied parameters. This could not be the case when the soil is already rich in nutrients or has a past record of cropping patterns that lead to soil enrichment up to a threshold, such as azotrophs and/or mycorrhizal associations (Saboor et al., 2021).

### Effect of bioinoculants on biochemical and physiological attributes of beetroot

For many years, research on the effect of mycorrhizae on chlorophyll and anthocyanin contents in various crops has been conducted. The research findings indicate that these biofertilizers enhance the different plant pigments to enhance immunity against different stresses and chlorophyll content to increase the number of photosynthates along with phosphorus and phosphatase contents (Lingua et al., 2013; Zare-Maivan et al., 2017; Begum et al., 2019a; Mahmud et al., 2021). Compared with the control plants, AMF-treated plants contain more chlorophyll because of antioxidants and supporting minerals, viz., N and Mg (to help in the biosynthesis and stabilization of necessary pigments). Due to this, more photosynthates (carbohydrates) as a carbon sink could enhance the photosynthesis rate. This is the first report on the effect of selected mycorrhizae on chlorophyll, anthocyanin, phosphate (in both shoot and root), and phosphatase content in beetroot. In this sequence, looking at the research findings, the total chlorophyll and anthocyanin contents were the highest, i.e., 25.346 and 27.912 mg FW^−*g*^ in the G_m_+A_l_+G_G_ treatment on beetroot, respectively, and the lowest in the A_l_ treatment, i.e., 21.452 and 24.028 mg FW^−*g*^, respectively (Supplementary Table 2). After looking into the phosphorus content in the shoot and root of the beetroot, the phosphorus content in the shoot and root was recorded as the highest in the Gm+Al+GG treatment, i.e., 1.007 and 2.849 (%), respectively. Following the same pattern, phosphorus content was the lowest, i.e., 0.737 and 1.134%, in the shoot and root of the beetroot, respectively. The phosphatase content (IU g-1 FW) in both acidic and alkaline forms was observed in all the treatments, and it was found that the Gm+Al+GG treatment had the highest phosphatase content in acidic and alkaline forms, i.e., 20.153 and 25.239, respectively. Again, the A_l_ treatment showed poor phosphatase content (acidic and alkaline forms), i.e., 17.221 and 21.743, respectively (Supplementary Table 2).

The analysis of the observed parameters indicates that among all the single and combined mycorrhizal treatments, the G_m_+A_l_+G_G_ treatment performed very well, and the A_l_ treatment performed poorly. Although, the findings suggest some good and poor performers of mycorrhizal treatment, the results may vary depending on soil health and crop-to-crop interaction (Pal and Pandey, 2015; Tsoata et al., 2015; Zhao et al., 2015). The enzyme phosphatase acts on the insoluble phosphate present in the soil and makes it available for plants. In this study, the Gm+Al+GG treatment showed the highest phosphorus and phosphatase contents, which may be because of the synergistic effect of all the AMF. All the treatments had higher values for all the observed parameters in comparison to the control (Supplementary Table 2).

### Effect of bioinoculants on stress physiological attributes of beetroot

**Figure 2:**
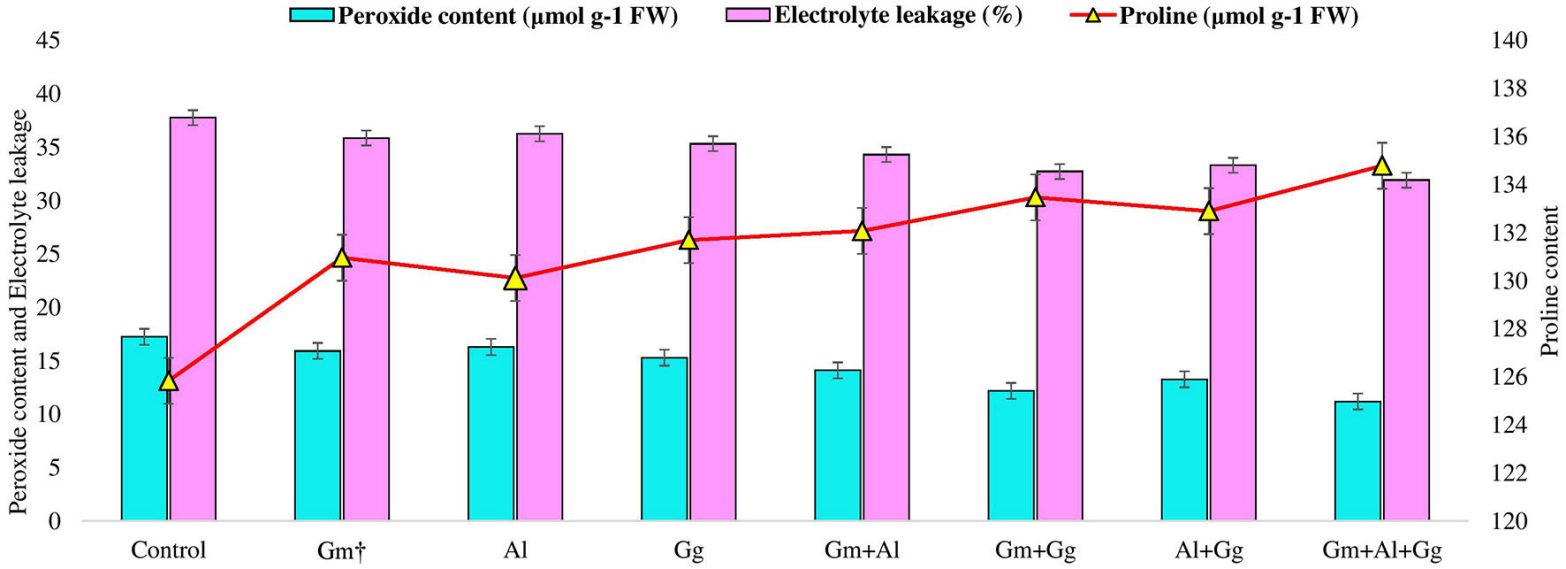
Peroxide content, electrolyte leakage, and proline content in control and treatment beetroot plant.

**Figure 3:**
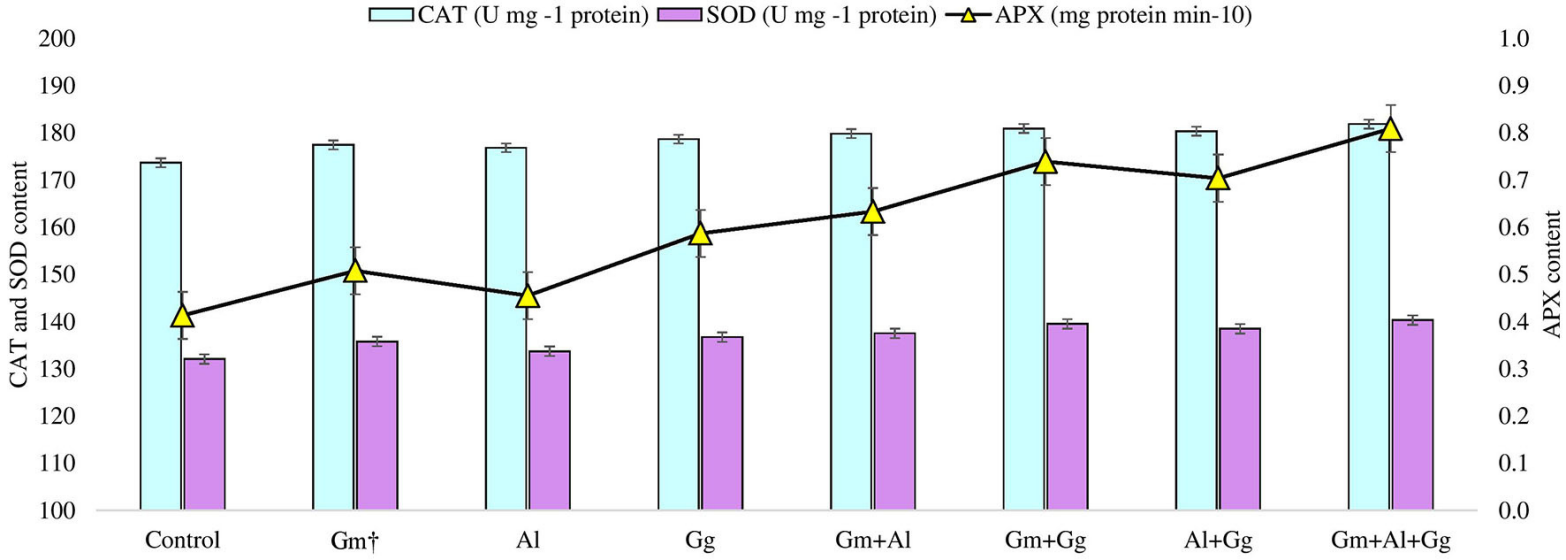
Catalase, superoxide dismutase, and ascorbate peroxidase content in control and treatment beetroot plant.

Under different biotic and abiotic stresses, peroxidation and electrolyte leakage are the major contributors to creating imbalances in the plant defense system (Talaat and Shawky, 2014; EshaghiGorgi et al., 2022; Haghighi and Saharkhiz, 2022). The application of mycorrhizae to study the level of peroxide content (μmol g^−1^ FW) and electrolyte leakage (%) on beetroot was recorded (Supplementary Table 3). In the experiment, we observed that the lowest peroxide and electrolyte leakage were found in the G_m_+A_l_+G_G_ treatment, i.e., 11.173 and 31.913, respectively, while the highest peroxide and electrolyte leakage were observed in the A_l_ treatment, i.e., 16.287 and 36.257, respectively (Figure 2). Along with this, we have also recorded the proline content (μmol g-1 FW) of the beetroot. It was observed that the highest level of proline was found in the G_m_+A_l_+G_G_ treatment, i.e., 134.788, and the lowest in the A_l_ treatment, i.e., 130.114 μmol g-1 FW (Figure 2). Furthermore, in this study, enzymatic activity was measured in beetroot, such as catalase (U mg-1 protein), ascorbate peroxidase (mg protein min-10), and superoxide dismutase (U mg-1 protein) (Supplementary Table 3). All three enzyme levels were the highest in the G_m_+A_l_+G_G_ treatment, i.e., 181.876, 0.809, and 140.333, respectively (Figure 3). Peroxidation and electrolyte leakage must be low to avoid any metabolic imbalance in the plant system (Chun et al., 2018). Therefore, we have given different treatments to record the levels of peroxidase and electrolyte leakage in control and treated beetroot. Our finding suggests that the use of biofertilizer and mycorrhizae in different combinations gives the best outputs, i.e., less peroxidase will be produced, leading to minimal lipid peroxidation and less electrolyte leakage. In the same way, proline content was measured. It is well known that proline is the major osmolyte, which plays an important role in maintaining the osmotic balance of the cell during various stresses, and different mycorrhizae boost the immune system of the plants by enhancing the level of proline in both unstressed and stressed conditions (Ortiz et al., 2015; Kulczyk-Skrzeszewska and Kieliszewska-Rokicka, 2022; Zahedi et al., 2022). Here, after treatment with mycorrhizae, it was found that proline concentration was increasing with the increasing mycorrhizal combination. The probable explanation for this could be that the single selected mycorrhiza could induce the production of proline minimally due to several reasons (Jeevan Kumar et al., 2015). However, when the mycorrhizal combination meets together, they might be associating symbiotically with plant roots, leading to the induction of genes to produce more proline in plants. There are various metabolic processes, such as photosynthesis and respiration, in the plant system during which different reactive oxygen species are produced. Reactive oxygen species (ROS) are not always harmful. Their balanced amount of ROS in the plant system is essential for successful metabolic activity inside various tissues (Haghighi et al., 2022). Even in open environments, stresses are always there for plants; hence, ROS levels are high all the time and increase exponentially, which damages biomolecules. In our findings, we have measured the levels of catalase, ascorbate peroxidase, and superoxide dismutase in beetroot plants in control (no treatment) and treatment conditions. We found that the levels of all three enzymes were the highest in the G_m_+A_l_+G_G_ treatment and the lowest in the A_l_ treatment (Figure 3). Previous studies also support our finding that mycorrhizal treatment enhances the level of these enzymes in different conditions to tackle the adverse effect of ROS (Jeevan Kumar et al., 2015; Ait-El-Mokhtar et al., 2022; Li et al., 2022; Naseri Rad and Naseri, 2022; Razvi et al., 2022). All the treatments showed more value than the control, indicating that no selected mycorrhiza is implicated in negative impacts on the beetroot.

### Effect of bioinoculants on the mineral content of beetroot

The treatments of various mycorrhizae have been given to study the effect of mycorrhizae on mineral uptake in plants that affects plant growth and development is well-known for many years. However, their implementations in agroecosystems have not been much explored. This study has shown the effect of mycorrhizal treatment in single and combination on the beetroot nutrient profile (Table 3). We analyzed the calcium, magnesium, potassium, iron, and manganese content (mg/100 g FW) in beetroot under control and treatment with different combinations of mycorrhizae. The best result was found in the G_m_+A_l_+G_G_ treatment in terms of nutrient uptake, such as potassium, calcium, magnesium, iron, and manganese in beetroot, while the least was observed in the Al treatment (Table 3). Our finding suggests that AMFs are very helpful in nutrient uptake from the soil, which will be available for plants. These minerals lead to biomass enhancement during the developmental process of the plants. In previous studies, single and combinatorial effects of mycorrhizae have been explored that strongly suggests that these mycorrhizae could be used as a biofertilizer (Liu et al., 2021; Rodrigues et al., 2021; Zhao et al., 2021; Ortas and Bilgili, 2022; Paredes-Jácome et al., 2022; Tereucán et al., 2022).

**Table 3:** Effect of bioinoculants on mineral contents of *Beta vulgaris*.

**Parameters → **	**K (mg/100 g FW)**	**Ca (mg/100 g FW)**	**Mg (mg/100 g FW)**	**Fe (mg/100 g FW)**	**Mn (mg/100 g FW)**

**Treatments** ↓					
Control	231.677 ± 1.182^f^	20.742 ± 1.27^e^	29.624 ± 0.661^e^	0.959 ± 0.118^e^	0.425 ± 0.844^d^
G_m_†	234.546 ± 0.967^d^	23.424 ± 1.02^c^	33.246 ± 1.018^cd^	1.293 ± 0.114^c^	0.484 ± 0.077^bcd^
A_l_	233.971 ± 0.785^e^	22.462 ± 1.57^d^	32.524 ± 1.068^d^	1.104 ± 0.172^d^	0.456 ± 0.052^cd^
G_G_	235.592 ± 2.215^cd^	24.018 ± 1.46^bc^	33.722 ± 1.153^bcd^	1.381 ± 0.125^c^	0.587 ± 0.044^ab^
G_m_+A_l_	235.501 ± 1.165^bc^	24.242 ± 1.18^b^	34.322 ± 0.924^ab^	1.564 ± 0.073^b^	0.508 ± 0.064^bcd^
G_m_+G_G_	237.561 ± 2.602^b^	25.194 ± 2.55^b^	34.992 ± 0.828^ab^	1.689 ± 0.135^ab^	0.572 ± 0.114^ab^
A_l_+G_G_	236.549 ± 1.683^bc^	24.876 ± 1.88^b^	34.878 ± 1.351^ab^	1.635 ± 0.101^ab^	0.521 ± 0.113^bcd^
G_m_+A_l_+G_G_	240.335 ± 1.257^a^	25.492 ± 1.05^a^	35.506 ± 1.128^a^	1.761 ± 0.075^a^	0.662 ± 0.093^a^
LSD (*P* ≤ 0.05)	2.057	1.441	1.334	0.152	0.108
ANOVA (Biermann, 1981; Lazǎr et al., 2021)	13.051	9.991	16.520	29.782	4.168

^†^G_m_, *Glomus mosseae*; A_l_, *Acaulospora laevis*; G_G_, *Gigaspora gigantean*. ±, Standard deviation; LSD, least significant difference test; FW, Fresh Weight.

### Gene expression analysis

**Figure 4:**
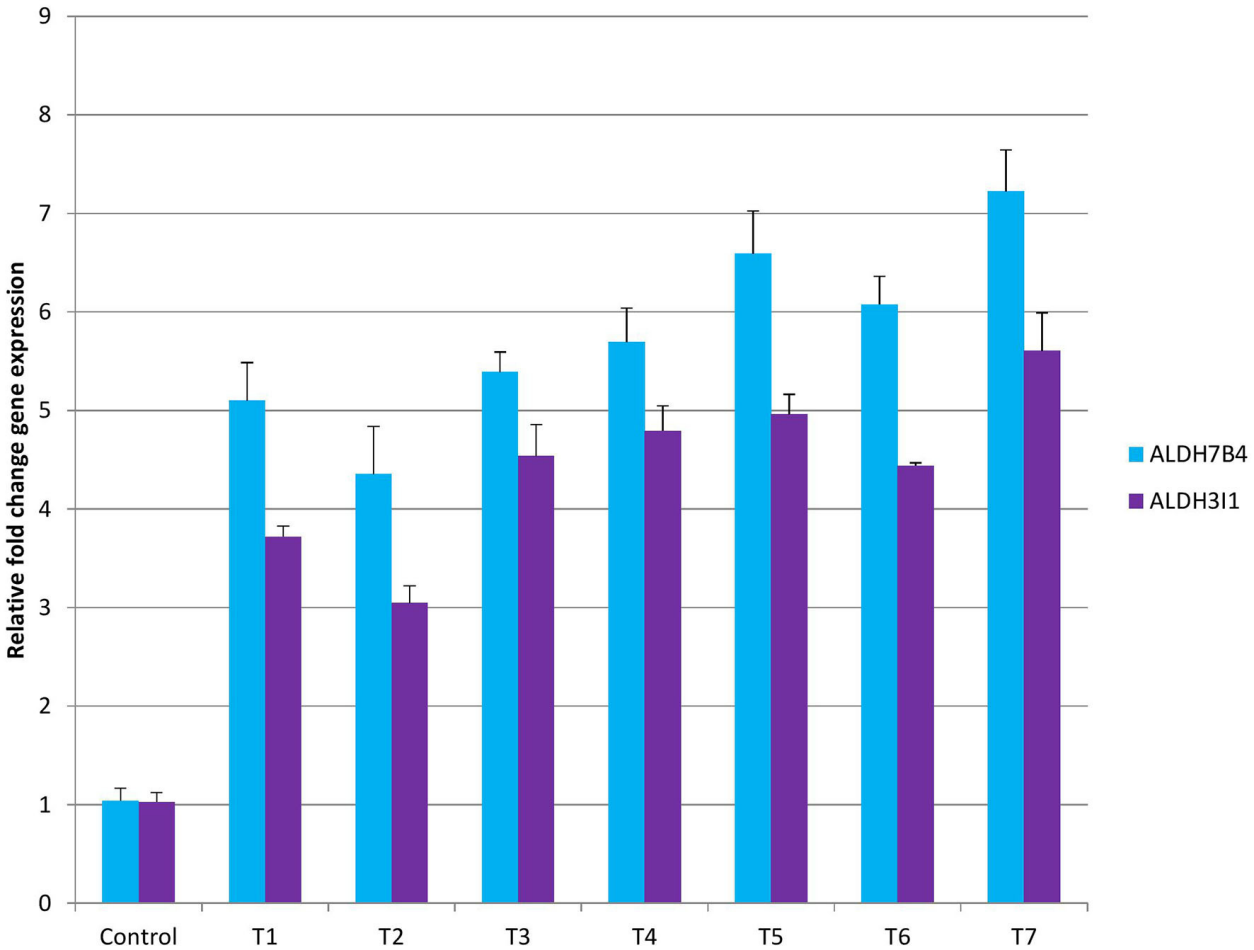
Gene expression analysis with ALDH7B4 and ALDH3I1 primers in control and treatment beetroot plant.

Gene expression analysis was performed using ALDH7B4 and ALDH3I1 primers. With the findings of biochemical analysis, it was evident that different treatments had variable responses on peroxide content, proline, catalase, ascorbate peroxidase, and superoxide dismutase levels. Furthermore, to validate the findings, we have performed aldehyde dehydrogenase gene expression that could lead to the lowering of peroxide content. In control beetroot plants, the ALDH7B4 gene was minimally expressed, while in all treatments, a different fold change in gene expression was observed (Figure 4). Among all treatments, the least gene expression was found in T2 (4.3 X) and the highest in T7 (7.2 X). The relative fold change of gene expression in T1 was 5.1X, T2 was 4.3X, T3 was 5.3X, T4 was 5.6X, T5 was 6.5X, T6 was 6.07X, and in T7, it was 7.2X. The chronology of treatment effects on *ALDH7B4* gene expression was C <T2 <T1 <T3 <T4 <T6 <T5 <T7. The second gene, ALDH3I1, was analyzed for gene expression. The least expression was observed in control plants (1X), followed by T2 (3.7X), while the highest gene expression was observed in T7 (5.6X). The relative fold change of gene expression in T1 was 3.7 X, T3 was 4.5 X, T4 was 4.7 X, T5 was 4.9 X, and in T6, it was 4.4 X. The fold change in T6 and T3 was almost equal (4.4 and 4.5, respectively). The chronology of treatment effects on ALDH3I1 gene expression was C <T2 <T1 <T6 <T3 <T4 <T5 <T7 (Figure 4). Aldehyde dehydrogenases are well known for their crucial role in aldehyde detoxification and scavenging ROS in plants (Kotchoni et al., 2006; Bhantana et al., 2021). In this study, upon various combinations of mycorrhizae treatment on beetroot plants, we observed different parameters such as peroxide content, proline, catalase, ascorbate peroxidase, and superoxide dismutase concentration (Supplementary Table 3). Begum et al. (2019b) studied the effect of AMF inoculation on catalase, peroxidase, and SOD synthesis against drought tolerance in maize. Similarly, the effect of AMF on antioxidants and their ROS scavenging capacity has been reported in the literature (Evelin et al., 2019). In our study, after mycorrhizal treatment, the peroxide level was observed to be higher in the control plants than in all the treated plants. The levels of proline, catalase, ascorbate peroxidase, and SOD were lowest in control plants, whereas in treated plants, the levels were significantly different and higher than in controls (Supplementary Table 3). Aldehyde dehydrogenases regulate the ROS level in organisms. Therefore, to know the probable reason for high levels of ROS in control and less ROS in treatments, we have analyzed the *ALDH7B4* and *ALDH3I1* gene expression. With the qRTPCR results, it is evident that different AMF treatments had different levels of effects on gene expression. The best-performing AMF combination was T7, which resulted in the highest *ALDH7B4* and *ALDH3I1* gene expression. This analysis supports our biochemical findings, where coherent results were obtained.

With the morphological, biochemical, and molecular analyses of the effect of different AMFs alone and in combinations on beetroot crops, the present study showed that AMFs are very effective for higher shelf life, higher production, and better resistance capacity. Therefore, with the current scenario of an increasing global population, high demand for foods, decreasing agricultural land area, high demand for organic foods, and environmental protection, the application of mycorrhizae for agricultural production is the best alternative to agrochemicals.

## Conclusion

The AMF treatment appears to be promising for beetroot production. In this direction, the AMF seems to be in a position to increase plant development, yield, and quality of beetroot. The optimum values for the biochemical and morphological traits have been based on the treatment with *G. mosseae, G. gigantean*, and *A. laevis*. The gene expression analysis validated the conclusions drawn based on morphological and biochemical data that the Gm+Al+GG consortium was the best among all combinations. Overall, our study could be a guide to exploring more microbes and mycorrhizal fungi for profitable beetroot production.

## Data availability statement

The original contributions presented in the study are included in the article/Supplementary material, further inquiries can be directed to the corresponding author.

## Author contributions

VY, DK, RJ, JS, AK, and AR have performed morphological, biochemical, and molecular work. RB, JS, GM, BV, and KJ have analyzed the data and performed the statistical analysis. AS and SK assisted in the proofreading of the MS. VY, DJ, and MR have written the MS and finalized it. All authors contributed to the article and approved the submitted version.
