# Supplementary material for: Mycorrhizae set the stage for plants to produce a higher production of biomolecules and stress-related metabolites: a sustainable alternative of agrochemicals to enhance the quality and yield of beetroot (Beta vulgaris L.)
Source: Front Microbiol. 2023 Jul 3;14:1196101. doi: 10.3389/fmicb.2023.1196101 (PMC10352028; doi:10.3389/fmicb.2023.1196101)
Supplement: Supplementary file 1 [file Table_1.docx]

| **Supplementary table 1: Effect of Bioinoculants on Morphological and Food storage parameters of  *Beta vulgaris*** | | | | | | | | |
| --- | --- | --- | --- | --- | --- | --- | --- | --- |
| **Parameters →** | **Shoot length (cm)** | **Root weight (g)** | **Shelf Life (days)** | **Root Circumference (cm)** | **Protein**  **(mg/100mg**  **FW)** | **Carbohydrate**  **(mg/100mg**  **FW)** | **AM spore number** | **AM root colonization (%)** |
| **Treatments ↓** |  |  |  |  |  |  |  |  |
| **Control** | 30.280±2.385^e^‡ | 237.214±6.552^g^ | 9.200±0.836^g^ | 19.610±0.751^f^ | 15.831±1.882^f^ | 9.959±0.736^g^ | 0.000±0.000^g^ | 0.000±0.000^g^ |
| **G_m_†** | 41.088±3.134^d^ | 254.889±4.462^e^ | 10.800±0.836^f^ | 20.810±0.929^d^ | 22.764±1.805^d^ | 10.831±0.432^ef^ | 33.800±2.387^f^ | 21.200±6.572^e^ |
| **A_l_** | 40.118±1.769^d^ | 248.716±3.241^f^ | 10.200±1.095^fg^ | 20.310±1.323^ef^ | 20.841±1.104^e^ | 10.142±0.935^f^ | 32.600±2.408^f^ | 16.800±2.387^f^ |
| **G_G_** | 45.426±1.808^c^ | 260.324±2.645^e^ | 11.400±1.101^de^ | 21.210±0.591^d^ | 24.894±1.135^d^ | 11.283±0.585^de^ | 35.800±4.147^f^ | 24.600.2±3.209^d^ |
| **G_m_+A_l_** | 47.148±2.057^bc^ | 272.674±3.236^d^ | 12.400±0.894^cd^ | 22.420±0.888^c^ | 26.621±1.183^d^ | 12.041±0.339^cd^ | 37.400±2.607^de^ | 26.600±43.107^c^ |
| **G_m_+G_G_** | 48.974±2.002^b^ | 297.932±5.451^b^ | 14.200±0.447^ab^ | 23.920±0.556^b^ | 33.308±1.751^b^ | 12.518±0.871^b^ | 47.200±5.631^b^ | 34.800±4.147^b^ |
| **A_l_+G_G_** | 46.086±1.442^bc^ | 287.768±5.085^c^ | 13.400±1.141^bc^ | 23.340±0.464^b^ | 30.056±1.177^c^ | 12.181±0.641^bc^ | 41.400±3.646^c^ | 32.600±4.393^b^ |
| **G_m_+A_l_+G_G_** | 51.974±3.149^a^ | 306.102±5.107^a^ | 14.800±0.836^a^ | 24.320±0.679^a^ | 38.092±1.979^a^ | 13.352±0.986^a^ | 55.200±3.834^a^ | 37.800±5.891^a^ |
| **LSD** (*P*≤0.05) | 2.957 | 5.981 | 1.196 | 1.049 | 1.989 | 0.933 | 4.444 | 2.161 |
| **ANOVA** (7, 32) | 42.867 | 140.882 | 22.907 | 23.361 | 106.529 | 13.488 | 110.144 | 41.552 |
| †G_m_- *Glomus mosseae*, A_l_- *Acaulospora laevis*, G_G_- *Gigaspora gigantean*  ±- Standard deviation; ‡values in column followed by the same letter are not significantly different; p≤0.05- LSD (least significant difference test); FW- Fresh Weight | | | | | | | | |

| **Supplementary table2: Effect of Bioinoculants on Biochemical and Physiological attributes of *Beta vulgaris*** | | | | | | |
| --- | --- | --- | --- | --- | --- | --- |
| **Parameters →** | **Total Chlorophyll**  **(mg FW^-g^)** | **Total Anthocyanin**  **(mg FW^-g^)** | **Phosphorous content**  **%** | | **Phosphatase**  **(IU g^-1^ FW)** | |
| **Treatments ↓** |  |  | **Shoot** | **Root** | **Acidic** | **Alkaline** |
| **Control** | 20.892±0.902^e^ | 22.814±0.534^d^ | 0.637±0.111^f^ | 0.982±0.166^f^ | 16.681±0.806^g^ | 20.973±0.494^f^ |
| **G_m_†** | 22.812±1.023^c^ | 24.846±0.721^c^ | 0.769±0.102^d^ | 1.206±0.407^de^ | 17.994±0.477^e^ | 22.057±0.471^d^ |
| **A_l_** | 21.452±1.223^de^ | 24.028±0.381^c^ | 0.737±0.131^e^ | 1.134±0.273^ef^ | 17.221±0.281^f^ | 21.743±0.453^ef^ |
| **G_G_** | 23.164±1.001^bc^ | 25.018±0.797^c^ | 0.819±0.066^cd^ | 1.622±0.572^cd^ | 18.195±0.897^d^ | 23.383±0.527^c^ |
| **G_m_+A_l_** | 23.834±0.926^bc^ | 26.082±0.547^b^ | 0.879±0.034^bc^ | 1.847±0.562^bc^ | 18.644±0.588^cd^ | 24.079±1.244^b^ |
| **G_m_+G_G_** | 24.866±1.056^b^ | 27.239±1.098^a^ | 0.991±0.062^b^ | 2.234±0.248^b^ | 19.665±0.629^b^ | 24.852±0.688^ab^ |
| **A_l_+G_G_** | 24.212±0.857^b^ | 27.138±0.899^a^ | 0.924±0.047^bc^ | 2.004±0.351^bc^ | 19.305±0.409^bc^ | 24.514±0.732^ab^ |
| **G_m_+A_l_+G_G_** | 25.346±1.968^a^ | 27.912±0.845^a^ | 1.007±0.187^a^ | 2.849±0.537^a^ | 20.153±0.703^a^ | 25.239±0.957^a^ |
| **LSD** (*P*≤0.05) | 3.014 | 0.978 | 0.132 | 0.537 | 0.815 | 0.958 |
| **ANOVA** (7, 32) | 8.987 | 26.932 | 7.821 | 11.477 | 18.132 | 22.738 |
| †G_m_- *Glomus mosseae*, A_l_- *Acaulospora laevis*, G_G_- *Gigaspora gigantean*  ±- Standard deviation; ‡values in column followed by the same letter are not significantly different;  p≤0.05- LSD (least significant difference test); FW- Fresh Weight | | | | | | |

| **Supplementary table 3: Effect of Bioinoculants on Stress related attributes of *Beta vulgaris*** | | | | | | | |
| --- | --- | --- | --- | --- | --- | --- | --- |
| **Parameters →** | **Peroxide content (μmol g^-1^ FW)** | **Electrolyte leakage (%)** | **Proline**  **(μmol g^-1^ FW)** | **Enzymatic activity** | | | |
| **Treatments ↓** |  |  |  | **Catalase**  **(U mg^-1^ protein)** | **Ascorbate peroxidase**  **(mg protein min^-10^)** | | **Superoxide dismutase**  **(U mg^-1^ protein)** |
| **Control** | 17.239±1.078^a^‡ | 37.773±1.483^a^ | 125.830±1.882^f^ | 173.677±1.575^f^ | | 0.413± 0.077^g^ | 132.081±1.246^g^ |
| **G_m_†** | 15.914±0.707^b^ | 35.857±1.592^b^ | 130.958±1.794^de^ | 177.456±1.093^de^ | | 0.508±0.062^ef^ | 135.794±1.811^e^ |
| **A_l_** | 16.287±1.051^b^ | 36.257±1.075^ab^ | 130.114±2.455^e^ | 176.851±1.469^e^ | | 0.455±0.055^f^ | 133.705±0.785^f^ |
| **G_G_** | 15.287±0.516^bc^ | 35.322±0.762^bc^ | 131.692±1.463^cd^ | 178.679±0.888^cd^ | | 0.587±0.044^de^ | 136.733±0.888^bde^ |
| **G_m_+A_l_** | 14.097±1.243^cd^ | 34.314±0.795^cd^ | 132.068±1.102^bcd^ | 179.855±1.071^bc^ | | 0.633±0.062^cd^ | 137.537±1.018^cd^ |
| **G_m_+G_G_** | 12.173±1.144^ef^ | 32.722±0.899^ef^ | 133.472±1.272^ab^ | 180.925±0.896^ab^ | | 0.739±0.091^ab^ | 139.537±0.411^ab^ |
| **A_l_+G_G_** | 13.257±1.095^de^ | 33.313±0.911^de^ | 132.894±1.313^bc^ | 180.359±0.762^b^ | | 0.704±0.079^bc^ | 138.505±1.218^bc^ |
| **G_m_+A_l_+G_G_** | 11.173±0.882^f^ | 31.913±0.622^f^ | 134.788±0.866^a^ | 181.876±0.905^a^ | | 0.809±0.1023^a^ | 140.333±1.254^a^ |
| **LSD** (*P*≤0.05) | 1.064 | 1.201 | 1.355 | 1.438 | | 0.095 | 1.278 |
| **ANOVA** (3, 16) | 22.849 | 22.480 | 16.360 | 28.238 | | 18.076 | 38.677 |
| †G_m_- *Glomus mosseae*, A_l_- *Acaulospora laevis*, G_G_- *Gigaspora gigantean*  ±- Standard deviation; ‡values in column followed by the same letter are not significantly different;  p≤0.05- LSD (least significant difference test); FW- Fresh Weight | | | | | | | |
